# Predictors of the Need for Permanent Pacemaker Implantation After Surgical Aortic Valve Replacement with a Biological Prosthesis and the Effect on Long-Term Survival

**DOI:** 10.3390/jcdd11120397

**Published:** 2024-12-11

**Authors:** Ivo Deblier, Karl Dossche, Anthony Vanermen, Wilhelm Mistiaen

**Affiliations:** 1 Department Cardiovascular Surgery, ZNA Middelheim Hospital, 2020 Antwerp, Belgium; ivo.deblier@zna.be (I.D.); karl.dossche@zna.be (K.D.); anthony.vanermen@zna.be (A.V.); 2 Faculty of Medicine & Health Sciences, University of Antwerp, 2610 Antwerp, Belgium

**Keywords:** surgical aortic valve replacement, permanent pacemaker implant, predictor, survival

## Abstract

The need for a permanent pacemaker (PPM) implantation after surgical aortic valve implantation (SAVR) is a recognized postoperative complication, with potentially long-term reduced survival. From 1987 to 2017, 2500 consecutive patients underwent SAVR with a biological valve with or without concomitant procedures such as CABG or mitral valve repair. Mechanical valves or valves in another position were excluded. Univariate and multivariate analyses were performed. The need for PPM implantation was documented in 2.7% of the cases. Patients with a postoperative PPM were older and had higher risk scores and a higher comorbid burden. Its predictors were a prior SAVR (odds ratio of 5.38, *p* < 0.001), use of a Perceval valve (3.94, *p* = 0.008), prior AV block 1–2 (2.86, *p* = 0.008), and pulmonary hypertension (2.09, *p* = 0.017). The need for PPM implantation was associated with an increased need for blood products, a prolonged stay in the ICU, and an increased 30-day mortality (2.5% vs. 7.0%, *p* = 0.005). The median survival decreased from 117 (114–120) to 90 (74–105) months (*p* < 0.001). The implantation had no significant effect on the freedom of congestive heart failure. The need for a PPM implant is not a benign event but might be a marker for a more severe underlying disease. Improving surgical techniques, especially with the Perceval rapid deployment valve, might decrease the need for a PPM implant.

## 1. Introduction

Aortic valve stenosis is the most commonly diagnosed structural heart valve disease, with a prevalence of 2 to 4% in the adult patient population [1]. Surgical aortic valve replacement (SAVR) is the oldest symptomatic relief and life-prolonging treatment for symptomatic aortic valve disease. Even in truly asymptomatic patients with severe aortic valve stenosis and preserved left ventricular function, early SAVR had a better clinical outcome compared to medical management with watchful waiting [2]. Postoperative conduction defects (CDs), such as high-degree atrioventricular (AV) blocks necessitating permanent pacemaker (PPM) implantation, have been recognized as serious complications after SAVR in 2–6% of the patients [3,4,5]. In isolated SAVR, the need for PPM implantation can be as low as 1.5%, and in a significant part of the patients, the conductivity can recover [6]. The reason for these postoperative conduction defects is the close proximity of the AV node and the bundle of His to the aortic valve annulus and the left ventricular outflow tract. This makes the cardiac conduction system vulnerable to injury during the operation. Sutures around the commissure between the right and non-coronary cusps should not involve the tissues of the membranous interventricular septum. Furthermore, ischemia, inflammation, edema, and the protrusion of the remaining calcific noduli could be involved [1,3,5,6,7]. Bioprosthetic valves with an extended component below the aortic annulus, such as with transcatheter and rapid deployment valves [8], exert an increased compression on the conduction system. These prosthetic valves, as well as the subannular implantation depth, are associated with markedly increased risks for the need for postoperative PPM implantation [9]. Parallel to this, sutureless and rapid deployment valves have been used in elderly and high-risk patients to lower the operative risk by shortening the aortic cross-clamp (ACC) and cardiopulmonary bypass (CPB) times as well as facilitating minimally invasive SAVR [7]. The need for PPM implantation might be higher compared to conventionally sutured valves [10]. The association between the need for a PPM implant and short-term adverse events as well as the long-term outcome is uncertain. Therefore, in this study, we aim to answer the following research questions: (1) What are the predictors of the need for implantation of PPM and (2) what are the long-term consequences of a postoperative PPM implantation?

## 2. Materials and Methods

This is a retrospective file study of 2500 patients who underwent SAVR with a biological prosthesis in the ZNA Middelheim General Hospital from 1 January 1987 to 6 July 2017. One hundred and two patients who had previously received a pacemaker were excluded. The preoperative and operative characteristics as well as the postoperative outcome are listed in Table 1. The definitions of preoperative factors, operative characteristics, and postoperative events have been described earlier [11,12]. Grade 1 and grade 2 preoperative atrioventricular blocks (AVBs) of the Wenckebach type, for which no immediate PPM implantation was needed, and a right as well as a left bundle branch block (RBBB and LBBB, respectively) were differentiated on an ECG. In all patients, a biological heart valve was implanted in the aortic position. A full sternotomy was performed in all patients, except for 183 individuals, in whom a partial sternotomy was the chosen access, according to the discretion of the surgeon. The CPB was installed, and the patient was cooled to 32 °C. After aortic cross-clamping, a warm blood cardioplegic solution was administered. Through a transverse aortotomy, the valve was inspected, the leaflets were resected, and the aortic annulus was decalcified, if necessary. After sizing, the valve was inserted with interrupted sutures, except for a Perceval device, for which only three temporary guiding sutures were used. A CABG, mitral valve repair, or another procedure was also performed if needed. After the removal of the cross-clamp, the patient was warmed up and the CPB was stopped. A control of the bioprosthetic function was performed by a transesophageal echocardiography. Provisional temporary pacemaker wires and chest tubes were installed, and the chest was closed. Except for 48 patients who received a sutureless Perceval device, all valves were conventionally sutured valves. Most of these sutured devices were of the Carpentier-Edwards perimount type [11]. The largest valve size for this prosthesis was 29. For the Perceval device, this was size XL. The outcome under investigation was the need for a PPM implant during the same admission period or within the first 30 days after SAVR. With the introduction of fast-track anesthesia in 1996, along with quicker extubation, the effect on the length of stay in the ICU and on the duration of mechanical ventilation was investigated only for the later patients. The main reason for a PPM implant was a persistent complete AV block. The statistical analysis included a univariate chi-square test for categorical variables and a Student’s *t*-test for continuous variables. A multivariate logistic regression analysis for the identification of predictors was performed by entering the significant factors identified by the univariate analysis. The effect of a PPM implant on long-term survival and freedom of congestive heart failure was evaluated by a Kaplan–Meier analysis with a log rank test (*p*-value), with a display of survival curves with 5-year and 10-year survival.

## 3. Results

In the 30 days following surgery, 417 patients (17.8%) had a new or worsening of a pre-existing conduction defect. Of these, 69 patients (2.8%) required PPM implantation. This procedure was indicated in 61 patients for a persistent total AV block and in 8 cases for slow atrial fibrillation or symptomatic bradycardia. Table 1 shows the comparison of the preoperative profile and operative characteristics of patients who needed a postoperative PPM implant versus those who did not. Except for age, all significant factors were cardiovascular in nature. Preoperative endocarditis had the largest effect, but the group was small. A comparable observation was made for patients who had undergone a prior SAVR. Pre-existing conduction defects as a group also had a major effect on the outcome. By subdividing these defects according to subtypes, AB block grade 1–2, but without the need for PPM implant, was significant. The effect of RBBB on the outcome was borderline significant, and the effect of LBBB was not significant. The need for postoperative PPM increased significantly from 1.8% before 2008 to 3.6% after 2008. This observation could be explained by the increase of several factors over time, which had an effect on the need for PPM implantation. These included the use of a Perceval device, which was not used in the earlier era, patient age greater than 75 years (which increased from 53.5% to 59.4%, *p* = 0.003), a prior AV block (which increased from 5.4% to 7.7%, with *p* = 0.023), and to a lesser degree, prior SAVR (which increased from 2.2% to 3.4%, with *p* = 0.057) and prior endocarditis (which increased from 1.7% to 3.7%, with *p* = 0.057). Systemic and pulmonary artery hypertension, congestive heart failure, and patient age greater than 75 also had a significant effect on the need for a PPM implant. Atrial fibrillation had a borderline effect. The mean age of patients needing a postoperative PPM implant was 75.6 ± 14.1 years, which was significantly higher than that of patients who did not need this implant (73.6 ± 14.4 years) (*p* = 0.014). However, with a Cohen’s D of −0.278, this difference was limited. For patients in need of a postoperative PPM implant, the severity of the aortic valve disease (mean TVG: 45.4 ± 16.1 mm Hg vs. 46.8 ± 15.2 mm Hg and AVA of 73.7 ± 29.9 mm^2^ vs. 73.2 ± 24.5 mm^2^) was comparable to those without a PPM implant, with *p* = 0.902. The Euroscore II of patients needing a postoperative PPM implant was significantly higher (10.5 ± 12.1% vs. 6.6 ± 7.7%, *p* = 0.013). The use of a sutureless and rapid deployment Perceval device as well as the largest size available for any bioprosthetic valve also resulted in a significant increase in postoperative PPM implantation. The choice for partial sternotomy had no significant effect on the need for a PPM (4/183 or 2.2% with *p* = 0.571).

Table 2 shows the adverse postoperative events and the need for resources associated with the need for a PPM implant. There were six patients who suffered from postoperative endocarditis. PPM implantation was not required for any of these patients. The need for a postoperative PPM implant was associated with an increased need for resources in terms of blood products and stay in the ICU (Table 2). Patients with the need for a PPM had 1.5 more units of packed cells (4.2 ± 3.5 vs. 2.7 ± 3.6 with *p* = 0.007), a stay of more than five days in the ICU (8.5 ± 15.8 days vs. 3.0 ± 7.1 days, *p* = 0.014), as well as postoperative stay (10.2 ± 8.5 days vs. 16.5 ± 10.9 days, *p* < 0.001). Mechanical ventilation, although more than 10 h longer, did not increase significantly (16.5 ± 53.6 h vs. 29.9 ± 56.5 h, with *p* = 0.116). Patients with the need for a postoperative PPM implant also had worse blood tests. For the lowest recorded postoperative hematocrit, this was 23.5 ± 3.2% vs. 24.8 ± 3.5%, with *p* = 0.007; for the lowest recorded arterial oxygen partial pressure, this was 74.4 ± 20.0 mm Hg vs. 85.2 ± 22.6 mm Hg, with *p* = 0.001; and for the highest recorded plasma glucose, this was 184 ± 36 mg% vs. 172 ± 48 mg%, with *p* = 0.033. All these effect sizes were considered small. Acute renal injury was worse in patients with the need for a PPM implant (Cohen’s D of less than 0.200). The mean increase in plasma creatinine was 0.84 ± 1.11 mg% vs. 0.38 ± 0.72 mg%, with *p* = 0.006 and a medium effect size (Cohen’s D of −0.638).

Table 3 shows the four independent predictors of the need for a PPM implant. Prior SAVR, the use of a Perceval sutureless device, and prior AB block were the three most important predictors. Although RBBB and the use of the largest valve size were identified as factors in univariate analysis, neither of these factors was identified as an independent predictor in the current series. The patients receiving the Perceval device were significantly older than those who received a Carpentier-Edwards device (75.2 ± 7.2 years vs. 81.4 ± 4.2 years, *p* < 0.001) and had more severe chronic kidney disease (with a GFR of 68.1 ± 22.2 vs. 55.6 ± 20.7 mL/min, *p* < 0.001). After a manual matching according to these two factors (48 Carpentier-Edwards valves vs. 48 Perceval devices), the effect on the need for postoperative PPM implantation was 1/48 (2.1%) for Carpentier-Edwards pericardial valves versus 5/48 (10.4%) for Perceval devices. Although this represented a five-fold increase, the sample size was too small to reach statistical significance (*p* = 0.092).

**Figure 1:**
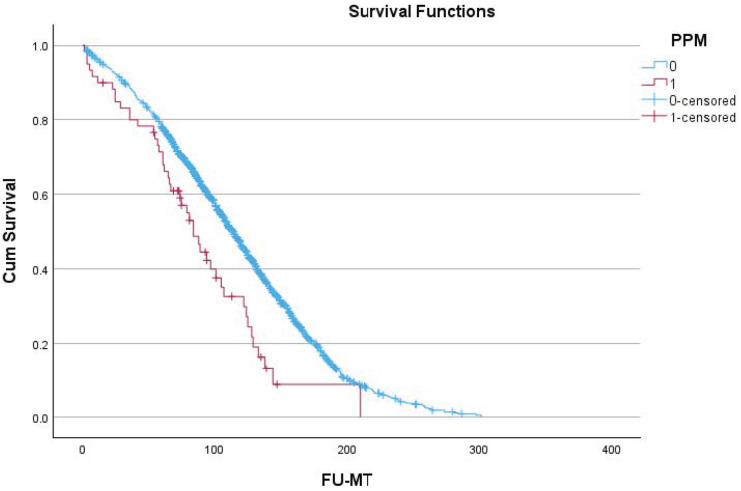
Survival of patients with (red line) and without PPM (blue) implant. FU-MT: follow-up in months; PPM: pacemaker; 0: patients without an implant; and 1: patients with an implant.

**Figure 2:**
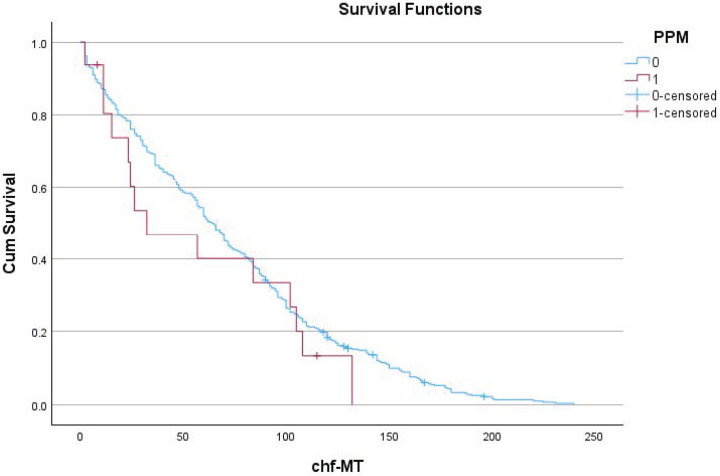
The need for PPM implantation had no significant effect on freedom for long-term CHF (*p* = 0.359). Chf-MT: freedom of congestive heart failure; PPM: pacemaker; 0: patients without an implant; and 1: patients with an implant.

Table 4 and Figure 1 show the effect of the need for a postoperative PPM implant on long-term survival. The need for a postoperative PPM implant had an effect on long-term survival. The mean survival time was 117 (114–120) months for patients without the need for a PPM implant. For patients needing a PPM, this was 90 (74–105) months, with *p* < 0.001. Figure 2 shows the effect of the need for a postoperative PPM implant on long-term congestive heart failure.

Number of patients at risk for survival for those with (+) and without (−) PPM (months)

$$\begin{array}{llllllllllllllll} & 12 & 24 & 36 & 48 & 60 & 72 & 84 & 96 & 108 & 120 & 132 & 144 & 156 & 168 & 190\\ \mathrm{PPM}\;(-) & 2230 & 2156 & 2063 & 1950 & 1818 & 1587 & 1380 & 1141 & 948 & 773 & 613 & 477 & 373 & 251 & 185\\ \mathrm{PPM}\;(+) & 54 & 52 & 47 & 45 & 40 & 33 & 24 & 17 & 13 & 11 & 6 & 2 & 1 & 1 & 0 \end{array}$$

Although the need for PPM implantation is a factor with an effect on survival, it could not be identified as an independent predictor of survival.

Number of patients at risk for heart failure for those with (+) and without (−) PPM (in months)

$$\begin{array}{llllllllllllllll} & 12 & 24 & 36 & 48 & 60 & 72 & 84 & 96 & 108 & 120 & 132 & 144 & 156 & 168 & 180\\ \mathrm{PPM}\;(-) & 365 & 328 & 290 & 255 & 228 & 188 & 164 & 129 & 98 & 81 & 59 & 51 & 35 & 21 & 15\\ \mathrm{PPM}\;(+) & 13 & 10 & 7 & 6 & 6 & 5 & 5 & 4 & 2 & 1 & 0 & 0 & 0 & 0 & 0 \end{array}$$

## 4. Discussion

In the current series, the most important independent predictor of the need for PPM implantation after SAVR was a grade 1 or grade 2 preoperative atrioventricular block of the Wenckebach type for which PPM implantation was not indicated at the moment of diagnosis. Furthermore, the rapid deployment Perceval device, a prior SAVR, and pulmonary artery hypertension were identified as predictors. The need for a PPM implant was associated with an increase in the most adverse postoperative events as well as an increased need for resources, such as blood products, a longer stay in the ICU and in the ward, and a significantly higher rate of mortality. However, these findings could be related to a significantly higher Euroscore II in patients needing a PPM. The need for a PPM implant was also associated with significantly reduced long-term survival but not with an increase in the long-term occurrence of congestive heart failure. For these reasons, the need for a postoperative PPM implant cannot be considered a benign event.

Prior conduction defects such as RBBB, LBBB, left anterior hemiblock, and AV blocks were diagnosed in our series in about 30% of the patients. This was somewhat below the observed 37.1% in an older series of 780 patients with a comparable age [13]. These defects were also commonly diagnosed in patients in older series with aortic valve disease [14,15] and could be a part of the degenerative and calcification process. Preoperative conduction defects were also identified as predictors of the postoperative need for PPM implants in earlier published series. In the first series of 2005, with 782 patients undergoing SAVR, these predictors included LBBB as well as a low ejection fraction and longer ACC and CPB times. The type of valve prosthesis had no significant effect [16]. In the second series, 15 of 207 (7.2%) of the patients required PPM implantation after SAVR. The predictors of this event in this series included preoperative first-degree AV block with and without left anterior hemiblock or intraventricular conduction defects. Other predictors were combined aortic and mitral valve surgery as well as postoperative cardiac arrest. The type of valve prosthesis had no effect. Of the patients who received a PPM, 70% remained dependent on a PPM during long-term follow-up [17]. In the third series, in which PPM implantation was needed in 3.2% of the patients, pre-existing LBBB and RBBB predicted the need for postoperative PPM implantation. There was no association with mortality [13]. In the fourth series, with 342 patients undergoing isolated SAVR, 29 patients (8.5%) needed PPM implantation. The only independent predictor was a preoperative conduction defect [15]. The presence of an AV 1 or 2 block was the only predictor of the need for PPM implantation in a series of 95 patients, of whom 10 (10.5%) needed a PPM implant. The presence of grade 3 calcification increased the sensibility of this observation [18]. In one series of 261 patients [14] with a mean age of 69 years, who were undergoing isolated SAVR, postoperative PPM implantation was needed in 8 (3%) patients. The need for PPM was similar in patients with and without preoperative conduction defects. Advanced aortic valve disease seemed of more importance for predicting the need for PPM implantation compared to prior conduction defects [14]. However, the number of patients was low and the statistical model could have been overfitted.

In one series, the need for PPM implantation after SAVR increased over time, which could be due to the introduction of the Perceval device, but no data were available to confirm this observation in this particular series [1]. However, the use of a Perceval device was identified as a predictor in the current series. The implantation of this valve requires shorter ACC and CPB times and was therefore introduced for elderly patients with a higher risk profile. Nevertheless, a higher postoperative rate of conduction defects [19] and a greater need for PPM implantation were observed [7,20]. Pre-existent conduction defects such as RBBB and AV blocks of grade 1 were predictive, together with older age, female gender, and large prosthesis size [21]. These observations were confirmed in another series, except for valve size [8]. This high rate of the need for PPM implantation could be lowered by adapting the surgical techniques. In one series with an elderly population undergoing isolated SAVR and with a high rate of reoperation, 10.5% of the patients required postoperative PPM implant because of complete AV blocks. Only RBBB was identified as a predictor of this event. Age was only significant in the univariate analysis [20]. In a series designed to study the effect of improved surgical techniques, prior AV conduction effects were not identified as a factor [7]. These surgical improvements included the avoidance of oversizing and gently pushing the white obturator of the Perceval device through the annulus [7,19,22,23]. The sizing policy of the prosthesis was also applied to the Intuity rapid deployment valve [24], indicating that this sizing issue is of major importance, irrespective of the design of the rapid deployment valve. Other technical adaptations were an adequate decalcification of the aortic annulus [7,19,23], higher positioning of the Perceval valve by placing the guiding sutures through the annulus rather than below it, and omitting the ballooning procedure after valve deployment [7,19]. By these measures, the high rate of postoperative PPM implantation of 16% could be lowered to 5.6% [7]. In several other series, this rate was reduced from 10–11% to 5–6%. This reduced rate of PPM implantation came closer to that of conventionally sutured valves with a comparable case mix [22,23].

The higher rate of PPM implantation after repeat cardiac surgery has been recognized decades ago. This was at almost 10% in a series of 558 consecutive patients who underwent repeat cardiac surgery, which involved a valvular procedure in over 50% of the patients [25]. In another relatively young patient group, SAVR resulted in the need for postoperative PPM implantation of 6.6%. Redo-SAVR was identified as one of its predictors. The other predictors were an additional mitral valve procedure for coexisting severe mitral valve insufficiency, a CABG, and a resection of a subaortic stenosis. Preoperative RBBB had an effect but only in the univariate analysis [26]. Prior cardiac surgery (18.0% vs. 8.7%) was also identified as a predictor of the need for postoperative PPM implantation in a nationwide survey of 5600 patients who underwent isolated SAVR. The need for PPM was at a level of 4% for the whole patient group. However, the effect of prior SAVR was more pronounced (14.2% vs. 4.8%), while the effect of prior mitral valve replacement was almost absent [1]. A comparison of redo-SAVR with a valve-in-valve TAVI and a similar risk score showed that the need for a PPM implant within 30 days was 5.9%, while for redo-SAVR, this was not significantly higher, at a level of 6.5% [27]. This suggests that both redo-procedures have similar morbidity and mortality.

Pulmonary artery hypertension was identified as a predictor of postoperative PPM implantation. In our series, this condition could only be routinely estimated by echocardiography in the last 1500 patients. It was present in 30% of the patients, higher than the 18% of patients in an older nationwide series of patients who were generally 5 years younger and were referred for either isolated SAVR or for TAVI [28]. Complications after SAVR or TAVI, such as stroke, bleeding, acute kidney injury, and the need for PPM implantation, were higher in patients who also had pulmonary artery hypertension. For SAVR, the need for PPM was 3.9% versus 4.8%, and for TAVI, this was 17.1% versus 18.5% [28]. Thus, it seems that the presence of pulmonary artery hypertension is a marker of a more advanced valve disease. This advanced valve disease could result in a higher postoperative complication rate, which includes the need for PPM. Pulmonary artery hypertension was also identified as a predictor of the need for PPM implantation within 3 months after cardiac surgery in general. During long-term follow-up, 61% of the patients remained dependent on the PPM. This indicated a 39% recovery rate of the conductivity [29]. In another series of patients undergoing isolated SAVR, the need for PPM implantation in the first 120 days after the operation was 3.8%. After the introduction of the TAVI program, this decreased to 1.5%. One of the independent predictors of the need for postoperative PPM was pulmonary artery hypertension [30].

Since postoperative conduction defects can be reversible, the timing of PPM implantation is important in order to avoid unnecessary procedures. A term of one [5] or two weeks after SAVR was proposed [6]. However, this policy needs to be confirmed. In one series, after implanting an Intuity rapid deployment valve, the median delay for implanting a PPM was 5.5 days, with an interquartile range of 4 to 7 days. The indications were a persistent complete AV block and a LBBB with a prolonged AV interval on electrophysiological studies [21]. A seven-day delay was recommended in case of potential reversible CDs, such as a second-degree “high-grade” AV block. In patients with a complete AV block and slow escape rhythm, recovery of the conduction was rarely observed. In these cases, the implantation of a PPM should occur in the short term. The decision should be at the discretion of the cardiologist [21]. As in the current series under investigation, patients needing a postoperative PPM implant also had more postoperative complications, such as atrial fibrillation, the need for prolonged ventilation, and acute renal injury. The need for a postoperative PPM implant was associated with reduced long-term survival [9,31], even after risk adjustment with the STS-PROM score [31]. This reduced long-term survival has been confirmed in a population with a mean age of 73 years, a good left ventricular function, and an associated CABG in 44% of the cases. A PPM after SAVR was implanted in 2.5% of the patients [32]. These observations could make it more difficult to disentangle the effects of a PPM from other conditions. The need for a PPM after any type of cardiac surgery has been identified as one of the independent predictors of reduced survival. A survival benefit was observed in patients with a restored rhythm after a PPM implant during follow-up. It should be noted that patients with a PPM but no structural heart disease have better survival than those with structural heart disease, such as valvular disease [33].

The long-term all-cause mortality as well as hospitalization for congestive heart failure in a nationwide series were higher in patients who needed a PPM implant after SAVR. For cardiovascular mortality, there seemed to be a trend. The mean age of these patients was almost identical: 69.7 years for patients without PPM and 69.8 years for patients with a PPM implant. Patients requiring PPM implantation had a higher degree of preoperative heart failure but a lower need for CABG. The other baseline characteristics were comparable to the current series [3]. Older series could not demonstrate an effect of PPM implantation on long-term survival, but the follow-up period might be too short since the differences become visible at 10 years. In older patient groups, competing causes of death might interfere with the observed effects. Conventional right ventricular pacing might be responsible for asynchrony with all its undesirable consequences [13,34]. In a low-risk nationwide population of almost 20,000 patients, a PPM implant within 30 days after SAVR resulted in a significant increase in the long-term incidence of hospitalization for congestive heart failure but not in all-cause mortality [4]. Permanent right ventricular pacing with adverse remodeling could negatively impact mortality and hospitalization for heart failure [4,35]. Postoperative PPM implantation in the current series was associated with an increased hospital stay, an increased stay in the ICU, an increased need for blood products, and, therefore, a higher cost. An increase in hospital stays was also observed in other series [3,13,31]. ICU stay increased by at least two days in another series [31].

## 5. Conclusions

In conclusion, the need for PPM implantation after SAVR was relatively low in this high-age patient group. The current observations suggest that the need for a PPM could result from surgical trauma and an ischemic burden during surgery. A preoperatively affected conduction system might promote further aggravation. This should not be surprising given the association between surgical manipulations at the fibrous skeleton of the aortic valve and the close vicinity of the conduction system. An inadequate decalcification of an aortic annulus with pressure from residual calcific noduli could be a contributing factor to the postoperative occurrence of a partial or complete AV block. The risk of injury might be increased during redo-SAVR. Furthermore, the use of sutureless and rapid deployment devices could also play a role, especially with oversizing. Pressure on the subannular tissue by radial forces during the expansion of bioprosthetic valves on the annulus could add to this effect. The identification of several preoperative predictors of postoperative PPM implantation and the association with a higher risk score in the current and prior series strengthens the impression that postoperative PPM dependency is a marker for underlying conditions, which, themselves, could reduce survival. The follow-up period needs to be long enough to confirm this view. The PPM implant was not identified as an independent predictor of long-term survival in the current and prior series. This observation seems to indicate that the need for a postoperative PPM implantation is a marker for a more complex disease.

### Strengths and Limitations

The strength of the current series is the size, the consecutive inclusion of the patients, and the long follow-up period. The retrospective nature is a limitation. This has been mitigated by the consecutive inclusion of the patients. Moreover, access to digitalized medical files allowed a detailed description of the patients. A propensity score analysis was not performed because of the large patient sample size, for which a multivariate regression analysis was more appropriate. Because of the high mean age at inclusion, many patients resided in a nurse facility during long-term follow-up, which might limit access to the data needed for follow-up. This is especially the case in patients in whom dementia has developed.

## Figures and Tables

**Table 1:** Preoperative and operative characteristics of patients with PPM.

Factor	Absent	%	Present	%	*p*-Value
Preoperative					
Endocarditis	58/2366	2.5	7/64	10.9	<0.001
Prior SAVR	58/2362	2.5	7/67	10.4	<0.001
Atrioventricular block 1–2	53/2274	2.3	12/158	7.5	<0.001
Conduction defects (all)	36/1473	2.1	28/682	4.1	0.005
Era: after 2008	22/1234	1.8	43/1189	3.6	0.006
Pulmonary artery hypertension	25/1034	2.4	23/449	5.1	0.007
Coronary artery disease	33/916	3.6	32/1514	2.1	0.027
Age > 75 years	21/1075	2.0	44/1375	3.2	0.050
Congestive heart failure	42/1819	2.3	23/610	3.8	0.053
Right bundle branch block	57/2258	2.5	7/132	5.3	0.055
Arterial hypertension	12/678	1.8	53/1747	3.0	0.084
Atrial fibrillation	44/1872	2.3	20/556	3.8	0.107
Chronic obstructive pulmonary disease	40/1732	2.3	22/630	3.5	0.112
Chronic renal dysfunction	50/2039	2.5	14/384	3.6	0.182
Need for urgent SAVR	46/1753	2.6	15/398	3.8	0.214
Prior CABG	58/2234	2.6	7/196	3.6	0.417
Male gender	31/1057	2.9	34/1375	2.5	0.486
Myocardial infarction	53/2049	2.6	12/377	3.2	0.510
LV ejection fraction < 50%	37/1450	2.6	11/363	3.0	0.612
Ischemic neurologic event	56/2100	2.7	8/331	2.4	0.755
Diabetes mellitus	51/1924	2.7	14/508	2.8	0.896
Carotid artery disease	52/1919	2.7	13/509	2.6	0.903
Operative					
Perceval implantation	60/2385	2.5	5/47	10.6	<0.001
Largest prosthesis size	56/2292	2.4	9/137	6.6	0.004
Concomitant CABG	33/980	3.4	32/1452	2.2	0.081
Cardiopulmonary bypass time > 120 min	28/1264	2.2	24/763	3.1	0.199
Mitral valve repair	61/2343	2.6	4/89	4.5	0.278
Procedure ascending aorta	57/2238	2.6	7/192	3.6	0.385
Aortic cross-clamp time > 60 min	19/1062	1.8	17/724	2.3	0.409
Incomplete revascularization	54/1926	2.8	7/214	3.3	0.697

CABG: coronary artery bypass graft; LV: left ventricular; and SAVR: surgical aortic valve replacement.

**Table 2:** Postoperative adverse events and need for resources for patients with PPM.

Factor	Absent	%	Present	%	*p*-Value
Need for resources					
Stay in intensive care unit > 1 day	7/1207	0.7	43/580	7.4	<0.001
Postoperative length of stay > 8 days	9/1268	0.7	48/783	6.1	<0.001
Renal replacement therapy	58/2327	2.5	7/92	7.6	0.003
Thrombocyte concentrate	33/1266	2.6	12/178	6.7	0.003
Reintervention	59/2359	2.5	6/80	7.5	0.006
>4 units packed cells	29/1137	2.6	16/309	5.2	0.018
Mechanical ventilation > 8 h	21/888	2.4	23/556	4.1	0.057
Plasma derivatives	27/1035	2.6	28/408	4.4	0.076
Adverse events					
Low cardiac output syndrome	51/2267	2.2	13/163	8.0	<0.001
Clinically relevant bleeding	53/2279	2.3	11/152	7.2	<0.001
Pulmonary complication	46/2117	2.2	18/314	5.7	<0.001
Acute renal injury	42/1984	2.1	23/444	5.2	<0.001
Delirium	41/1821	2.3	14/214	6.5	<0.001
Mortality	56/2302	2.4	9/128	7.0	0.002
Myocardial damage	61/2408	2.5	3/23	13.0	0.028
New or recurrent atrial fibrillation	30/1454	2.1	34/997	3.5	0.032
Thromboembolic event	59/2347	2.5	5/84	6.0	0.053
Complex ventricular arrhythmia	60/2335	2.6	4/95	4.2	0.328

**Table 3:** Predictors of PPM implant.

Predictor	Odds Ratio	95% CI	*p*-Value
Prior SAVR	5.38	2.09–13.87	<0.001
Perceval implant	3.94	1.44–10.77	0.008
Prior atrioventricular block 1–2	2.86	1.32–6.23	0.008
Pulmonary artery hypertension	2.09	1.14–3.69	0.017

CI: confidence interval and SAVR: surgical aortic valve replacement.

**Table 4:** Long-term survival with PPM versus without PPM.

Survival	Without PPM	N	With PPM	N
60 months	77.9 ± 0.9%	1818	67.7 ± 6.1%	40
120 months	46.0 ± 1.1%	773	29.8 ± 6.7%	11
180 months	17.9 ± 1.0%	185	None	

PPM: Permanent pacemaker and N: number of patients at risk.

## Data Availability

These results have been derived from a multipurpose database, from which several more publications will be derived. These data are not yet publicly available.
